# Recruitment and retention of women in a large randomized control trial to reduce repeat preterm births: the Philadelphia Collaborative Preterm Prevention Project

**DOI:** 10.1186/1471-2288-10-88

**Published:** 2010-09-29

**Authors:** David A Webb, James C Coyne, Robert L Goldenberg, Vijaya K Hogan, Irma T Elo, Joan R Bloch, Leny Mathew, Ian M Bennett, Erika F Dennis, Jennifer F Culhane

**Affiliations:** 1 Department of Adolescent Medicine, Children's Hospital of Philadelphia, 3535 Market Street, Philadelphia, Pa., 19104, USA; 2 Department of Psychiatry, University of Pennsylvania School of Medicine, 3535 Market Street, Philadelphia, Pa.,19104, USA; 3 Department of Obstetrics and Gynecology, Drexel University College of Medicine, 1525 Race Street, Philadelphia, Pa., 19104, USA; 4 Department of Maternal and Child Health, University of North Carolina at Chapel Hill, 421 Pittsburgh Street, Chapel Hill, NC, 29599, USA; 5 Department of Sociology, University of Pennsylvania, 3718 Locust Walk, Philadelphia, Pa., 19104; 6 Department of Nursing, Drexel University College of Nursing and Health Professions, 1520 Race Street, Philadelphia, Pa 19102, USA; 7 Department of Adolescent Medicine, Children's Hospital of Philadelphia, 3535 Market Street, Philadelphia, Pa., 19104, USA; 8 Department of Family Practice and Community Medicine, University of Pennsylvania Health System 3400 Spruce Street, 19104, USA; 9 Department of Neonatology, Children's Hospital of Philadelphia, 3535 Market Street, Philadelphia, Pa., 19104, USA; 10 Department of Pediatrics, University of Pennsylvania School of Medicine, 3535 Market Street, Philadelphia, Pa., 19104, USA

## Abstract

**Background:**

Recruitment and retention of patients for randomized control trial (RCT) studies can provide formidable challenges, particularly with minority and underserved populations. Data are reported for the Philadelphia Collaborative Preterm Prevention Project (PCPPP), a large RCT targeting risk factors for repeat preterm births among women who previously delivered premature (< 35 weeks gestation) infants.

**Methods:**

Design of the PCPPP incorporated strategies to maximize recruitment and retention. These included an advanced database system tracking follow-up status and assessment completion rates; cultural sensitivity training for staff; communication to the community and eligible women of the benefits of participation; financial incentives; assistance with transportation and supervised childcare services; and reminder calls for convenient, flexibly scheduled appointments. Analyses reported here: 1) compare recruitment projections to actual enrollment 2) explore recruitment bias; 3) validate the randomization process 4) document the extent to which contact was maintained and complete assessments achieved 5) determine if follow-up was conditioned upon socio-economic status, race/ethnicity, or other factors.

**Results:**

Of eligible women approached, 1,126 (77.7%) agreed to participate fully. Of the 324 not agreeing, 118 (36.4%) completed a short survey. Consenting women were disproportionately from minority and low SES backgrounds: 71.5% consenting were African American, versus 38.8% not consenting. Consenting women were also more likely to report homelessness during their lifetime (14.6% vs. 0.87%) and to be unmarried at the time of delivery (81.6% versus 47.9%). First one-month postpartum assessment was completed for 83.5% (n = 472) of the intervention group (n = 565) and 76% (426) of the control group. Higher assessment completion rates were observed for the intervention group throughout the follow-up. Second, third, fourth and fifth postpartum assessments were 67.6% vs. 57.5%, 60.0% vs. 48.9%, 54.2% vs. 46.3% and 47.3% vs. 40.8%, for the intervention and control group women, respectively. There were no differences in follow-up rates according to race/ethnicity, SES or other factors. Greater retention of the intervention group may reflect the highly-valued nature of the medical and behavior services constituting the intervention arms of the Project.

**Conclusion:**

Findings challenge beliefs that low income and minority women are averse to enrolling and continuing in clinical trials or community studies.

## Background

Randomized control trials (RCTs) are widely regarded as the most robust research designs for assessing the efficacy of medical and behavioral interventions [1,2]. However, in some cases, barriers pertaining to the recruitment and retention of patients for RCT studies are formidable, and may result in compromised execution of studies and prohibitive costs [3]. Perhaps most importantly, unanticipated recruitment or retention problems pose threats to study integrity, either in terms of generalizeability of the findings, or bias associated with selective attrition of intervention or control group subjects [3,4].

Successful recruitment and retention of sufficient numbers of subjects drawn from a diverse urban population may be especially challenging [5]. A lack of understanding of the purpose of medical research and research designs, general distrust of doctors and hospitals, and structural barriers stemming from lack of transportation, child care obligations, inflexible work schedules, and the hassles of everyday life have all been noted by some observers as potential problems that may undermine the recruitment and retention of subjects in RCT studies [3,4,6,7]. These problems are widely thought to be more prevalent in minority, low income populations, or those living in distressed neighborhoods, where services may be inferior, housing conditions tend to be poor, rates of crime are high, and mobility may be seriously limited [3,4,6,8]. Hence the potential for under-representation as a result of low recruitment or high attrition of disadvantaged, vulnerable populations warrants serious attention. Not surprisingly, a large body of research has emerged in an attempt to identify strategies to address this issue, including locating services and intervention sites where participants can be easily reached, providing appropriate and adequate incentives for enrollment and continued participation, and increasing the sensitivity and ability of staff to deal with cultural differences which may contribute to suspicion and reluctance of patients to consent or adhere to the study protocols [5,9,10].

While the conventional wisdom is that problems associated with the recruitment and retention of subjects for RCTs tend to disproportionately 'screen-out' minority, low income, or other residents facing difficult life circumstances, data which directly document the issue are surprisingly scant [6]. One systematic study of recruitment data pertaining to 70 health intervention studies in the U.S. led the researchers to conclude that, contrary to conventional wisdom, participation rates were not consistently lower for minority as opposed to non-minority persons [7]. As some observers have argued, factors such as the real and perceived value of what the study offers to the community and potential subjects themselves could outweigh or at least counterbalance those associated with inconvenience, accessibility, residential instability, fear, or suspicion [5,6].

Reported here are the recruitment and retention data pertaining to the Philadelphia Collaborative Preterm Prevention Project (PCPPP), a large randomized control trial involving Philadelphia resident women who previously delivered premature (< 35 weeks gestation) infants. Described in detail below, the study was designed to address an array of risk factors for future preterm delivery and thus reduce the rate of repeat preterm births in the intervention group. The protocols for recruitment were without regard to race/ethnicity or other socioeconomic factors, but a concerted effort was made to avoid as well as document any bias in enrollment or selective attrition, especially that associated with membership in minority or low socioeconomic status groups, the group most at-risk for preterm and repeat preterm births. The PCPPP study protocol called for continuous and regular risk assessments, including living conditions, and physical and mental health status of both intervention and usual care participants, for up to a two-year period from baseline enrollment. An advanced database system was developed to track the follow-up status and assessment completion rates, and it proved to be a critical tool for the purposes of cohort maintenance. The data captured by this system were particularly useful for documenting baseline recruitment and retention rates for an urban study population with a substantial proportion of low income and minority residents, and for exploring the extent to which these rates varied by socio-demographic and other relevant factors.

## Background: Study Rationale

Preterm birth (PTB) is an adverse pregnancy outcome which in the U.S. accounts for 65-75% of infant mortality and nearly 50% of long-term handicapping conditions in children [11,12]. Despite reductions in infant mortality over the past 40 years, the PTB rate has remained stable, and has been a persistent source of significant race/ethnic and socioeconomic disparities. Although the causes of PTB are not well-understood, a substantial number of cases occur in association with an inflammatory process marked by increased membrane production and elevated amniotic fluid levels of various proinflammatory cytokines, prostaglandins, and metalloproteases [13-16]. In many cases of PTB, a uterine infection is present, but in others, the origin of the inflammatory process is found elsewhere in the mother's body, or not at all. Infections associated with PTB include periodontal disease, urinary tract infections, bacterial vaginosis, pneumonia and influenza [17-20]. In addition to infection, many of the other risk factors for PTB are proinflammatory (e.g., smoking, obesity, depression and psychosocial stress), and are also associated with increased levels of systemic inflammatory markers (SIM) such as, IL-6, C-reactive protein and serum amyloid A [16,21]. These behavioral risk factors may act through a common inflammatory pathway leading to enhanced risk of PTB.

The hypothesis that seemingly disparate yet well-known risk conditions lead to PTB through a common physiological pathway may explain the persistence of population-based race/ethnic disparities, as well as the individual propensity for repetitive PTB. First, at the population level, these risk conditions are highly prevalent and tend to co-occur in socioeconomically disadvantaged populations. The co-occurrence of multiple risk factors associated with poverty and minority status may put entire groups of women at heightened risk for generalized systemic inflammation (SI) [21-24]. Secondly, at the individual level, the best predictor of PTB is a previous PTB, such that women with a previous PTB are at 2 to 6-fold greater risk for a second PTB, compared to women with a previous full term birth [25]. This suggests that the sources of individual physiological risk persist over time. Furthermore, the earlier the gestational age of any PTB, the greater the risk for a repeat PTB. In one completed progesterone trial, for example, more than 50% of women in the usual care group with a prior history of PTB at approximately 32 weeks gestational age (GA) had a subsequent PTB [26].

Virtually all interventions aimed at reducing PTB, including those that target the inflammatory pathway, have been conducted *during pregnancy *[12]. These include interventions to improve access to high quality prenatal care, bed rest, nutrition counseling, caloric or vitamin/mineral supplementation, smoking, drug or alcohol cessation programs, and treatment for pelvic infections. The delay of interventions until pregnancy is achieved may explain why most of the interventions have failed to reduce PTB, since once the inflammatory cascades are initiated, it may be too late to interrupt the processes. Furthermore, it may take weeks or months after the behavioral risk factor is modified to register consequent reductions in SI.

**Figure 1:**
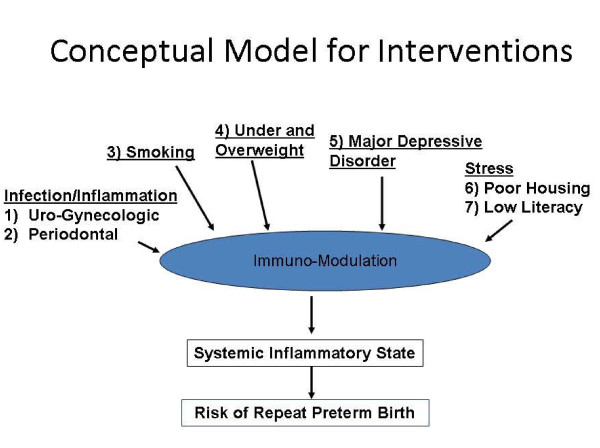
Conceptual Model for Interventions

For these reasons, we proposed a multifaceted behavioral risk reduction approach *during the interconceptional period *in a population of women who have already experienced a previous PTB at < 35 weeks gestation. The conceptual model for the intervention is depicted in Figure 1.

## Methods

The intervention plan was evidence-based, focusing on reduction of established salient risk factors for SI and thus a repeat premature delivery. Specifically, the aim was to reduce the effects of SI by lowering the risks associated with smoking, depression, infectious disease burden and maternal stress, and achieving an appropriate BMI. Assessment of risk for both the intervention and usual care group was ongoing and scheduled to occur at regular intervals (at 1, 6, 12, 18 and 24 months postpartum). The core protocol provided careful and comprehensive risk assessment, appropriate case-management and free treatment for the intervention group, while the Usual Care Group received only the assessment component. A descriptive outline of the core protocol is provided with some detail in Table 1. In brief, the intervention was evidenced-based, by focusing on the most salient social, behavioral and medical factors which have been shown to be associated with SI, or directly with an increased risk of PTB. As presented in Table 1 the interventions fell into six major categories: maternal infection, periodontal disease, exposure to nicotine (cigarette smoking), inadequate nutrition or abnormal body weight, maternal depression, and maternal stress. Note that in all cases both assessments and related interventions were conducted by qualified and experienced personnel.

**Table 1:** Outline of Core Protocol for PCPPP

	*Intervention*	*Usual Care/Controls*
**Risk factor**		

***Infection***	**Assessment**: Standard medical diagnostics for STD's and urogenital tract infections, including Bacterial Vaginosis (BV), Chlamydia, Gonorrhea, and Syphilis. Tests were conducted at the centralized clinical setting; all lab results were reviewed by the study-trained laboratory coordinator. **Treatment**: Follow-up and standard medical treatment for all conditions identified; supervised by the study medical team and free of charge.	**Assessment**: standard medical diagnostics for BV only.

***Periodontal Disease***	**Assessment: **Screening for clinical periodontal disease including soft tissue exam for oral cancer, Plaque Scores, Gingivitis Index Scores, Probing Pocket Depth, Bleeding Upon Probing, Clinical Attachment Level, and cementoenamel junction. Assessment was completed by the study registered dental hygienist. **Treatment: **Individually-tailored intervention including oral hygiene education and comprehensive clinical treatment for all conditions identified. Presence of periodontal disease was confirmed through x-ray and clinical exam by a DDM. Treatment for periodontal disease was provided by or under direct supervision of a periodontist/DDM, free of charge	**Assessment: **Same as for Intervention Group

***Smoking***	**Assessment: **Evaluation of smoking status during pregnancy and postpartum using standardized questionnaire. Questionnaire was administered by study staff. **Treatment: **Referral and follow-up for smokers, who were offered individually-tailored one-on-one cessation counseling and pharmacotherapies, including standard nicotine replacement therapy, and bupropion. One-on-one counseling was provided free of charge by smoking cessation counselor; pharmacotherapy was provided and prescribed by physician free of charge	**Assessment: **Same as for Intervention Group

***Inadequate Nutrition/Weight***	**Assessment: **Documentation of clinical, body weight/mass, including weight change, skin fold thickness and waist circumference; evaluation of diet and nutritional intake. The assessments were completed by a certified Dietician with a Master's degree in Human Nutrition. **Treatment: **Individually-tailored program of nutritional education and diet supplementation, including vitamins; food assistance if necessary; development of a weight loss program (including exercise), if appropriate.	**Assessment: **Same as for Intervention Group

***Major Depressive Disorder***	**Assessment: **Screening with Center for Epidemiological Studies of Depression Scale (CES-D ≥16) followed by a diagnostic interview with the Structured Clinical Interview for DSM Disorders (SCID) for those with a positive screen. The SCID was administered by appropriately trained study physician or social worker. **Treatment: **Participants who were diagnosed with current major depressive disorder were offered medial treatments comprised of cognitive behavioral therapy, antidepressant psychopharmacology, following manualized protocols, or the combination of the two treatments. Women who declined these therapies were offered supportive counseling and problem solving training delivered by clinical social workers in home visits.	**Assessment: **Same as for Intervention Group, although SCID was not performed for those who screened positive for depressive symptomatology

***Maternal Stress***		

*a. **Literacy***	**Assessments: **Short Test of Functional Health Literacy in Adults (inadequate and marginal: English and Spanish), Test of Adult Basic Education - Reading Locator (levels E&M; TABE). Questionnaire was administered by study staff. **Treatment: **An individually tailored learner-driven intervention model was utilized using a contextual adult educational curriculum focused on building skills for navigating hurdles to maternal-infant care and family management/economics. Adult literacy skills were developed through working individually with professional adult educators on specific challenges faced by the participants and selected by them.	**Assessment: **Same as for Intervention Group

*b. **Housing*** ***Instability***	**Assessment: **Comprehensive assessment of housing status and stability conducted by study staff. **Treatment: **Housing assistance, when appropriate, was provided in the form of cash grants for down payments or back rent, relocation services, or resolution of landlord/renter disputes, provided under the direction of a MSW, with experience in resolving housing-related issues.	**Assessment: **Same as for Intervention Group

To demonstrate the efficacy of the intervention, we carried out a randomized trial, recruiting Philadelphia resident women from the twelve hospitals providing 70% of the obstetric services in the region. Eligibility for enrollment was defined as follows: 1) delivery of live born singleton infant at <35 weeks of gestation; 2) English or Spanish speaking; 3) Philadelphia residency; and 4) *not *receiving operative sterilization before discharge from the hospital. During the postpartum hospital stay, project staff explained the study and attempted to obtain written informed consent. For those women who refused to participate, we obtained consent to collect pertinent medical records for the index pregnancy, the outcome of the next pregnancy via vital records linkage, and to conduct a much abbreviated survey to obtain data on important demographic, psychosocial and behavioral risk factors. Prior to the beginning of the study, based on individual birth record data for a historical six-year birth cohort, we calculated that approximately 1,670 <35 week singleton PTB's would occur during the 18-month enrollment period, and that 85% of the women would be eligible and agree to participate (n = 1,420). We also determined that 43% of the women would experience a subsequent live birth within the following 2-year period. In addition, of the women with a subsequent live birth, 50% of the second pregnancies were estimated to be <35 weeks gestation.

Ongoing data collection and risk assessment were an integral part of the study design with the objective of continuously identifying, referring and providing treatment for all women in the intervention group who had any of the risk factors associated with SI noted above. As reflected in Table 1, risk assessment/data collection visits for the Usual Care group closely mirrored that for the Intervention group, both in terms of the timing, follow-up period and scope of information collected. If a woman in either group became pregnant following the index birth, the protocol called for the last formal assessment/data collection time point to occur at 20 weeks gestation of the subsequent pregnancy, and for all relevant data pertaining to the period since childbirth to be abstracted from the medical chart. Regardless of when women were enrolled during the 18-month enrollment window, which began 11/01/2004, all risk assessments/data collection visits were scheduled to end on 9/1/2008 under the study protocol. Thus, women who enrolled after 9/1/2006 were not eligible for the full two-year follow-up visits and interventions.

### Randomization and Retention Strategies

Recruitment and enrollment was underpinned by an agreement with each of the twelve study hospitals who designated a contact person (usually someone on the delivery floor) who would check the delivery logs for eligible women, obtain a Health Insurance Portability Act (HIPAA) release form, and fax the form to the study outreach team at a pre-designated secure fax number. Study staffs were dispatched to the hospital to confirm eligibility and obtain the mother's consent and enroll her in the study.

Women who consented were then randomized into either the intervention or control group, stratified by gestational age of the infant, as follows. Study ID numbers were generated and stratified into two groups: (1) women delivering infants < 30 weeks gestation; and (2) those delivering infants ≥30 weeks gestation. Within each stratum, a computerized random sampling program was implemented in Stata and used to select 50% of Study ID numbers for the Intervention group, with the remaining 50% allocated to the Usual Care group. Randomization cards were created, stating either "Intervention Group" or "Usual Care Group," and were placed into sealed envelopes. On the outside of each sealed envelope, the corresponding Study ID number and gestational age group were noted. The envelopes were stored in a locked closet, supervised by the Study Manager. At the time of enrollment, prepared study materials designated for either intervention or usual care enrollees were signed out by Study Coordinators. When a study participant consented to participate in the study, the Study Coordinator selected the packet with the next consecutive Study ID number within the appropriate stratum (that is, depending on the gestational age of the infant at birth), and opened the sealed envelope to reveal the randomization card. The appropriate Study ID number and corresponding randomization group were then assigned to that study participant for the duration of the study.

Multiple overlapping strategies were employed to ensure maximum enrollment and retention rates. Project staff was given extensive cultural sensitivity training which prepared them to appropriately answer questions about the study and to address specific concerns of potential study participants. The benefits of the study to the community and to the participants were also fully explained. Because all infants of enrolled mothers were medically high risk, including at a high risk of an infant death, all interviewers received training in grief counseling and bereavement. Financial incentives were built into the study design and were explained to all potential participants at the time of enrollment. Progress toward recruitment goals was closely monitored and shared among staff, and a real-time response system for identifying and resolving recruitment problems was developed and implemented.

To maximize follow-up success, multiple levels of contact information were collected and recorded at the time of enrollment. Each participant completed an 'initial contact' form, which included her home telephone number, work number, cell phone number and complete address as well as telephone numbers and addresses of three close friends or relatives. This information was routinely updated at each study visit.

At the time of enrollment all participants were scheduled for a one month data collection visit to take place at a study clinic. Subsequent visits were scheduled in similar fashion according to the data collection protocol described above. A financial incentive of $40 was given for completing each visit. Reminder calls were placed a few days prior to each scheduled visit. All participants were carefully tracked using advanced database technology to determine their risk assessment/data collection status, which was shared with all project staff. Weekly progress reports were generated and reviewed by staff and weekly meetings were held with the PI to identify and resolve any difficulties with follow-up. Difficult to find (DTF) participants were identified and a protocol was developed to provide intensified follow-up activities and DTF cases were assigned to case managers, longtime community residents who had extensive experience locating and conducting health interviews with women in their community. These case managers often made multiple home visits, arranged childcare, transportation or personally escorted participants to their scheduled study visits.

Because all routine assessment visits for the intervention and control groups occurred at the main clinic site, all women were offered assistance with transportation and supervised child care services were made available at the site. Transportation assistance included reimbursement for public transportation, or parking costs, as well as cab fare. Every effort was made to schedule assessment visits at the convenience of participants, including evening and weekend hours.

### Analysis of Recruitment, Randomization and Retention Data

The analysis of recruitment and retention data presented here had several objectives: 1) to compare our recruitment projections to actual enrollment figures; 2) to explore the extent of recruitment 'bias', particularly as it pertains to under-enrollment of minority women, women from low socioeconomic status households, or otherwise disadvantaged families; 3) to validate the randomization process, by comparing those in the intervention group to those in the control group on a wide range of socio-demographic, health-related and other relevant characteristics; 4) to document the extent to which we were able to maintain contact and complete assessments for both intervention and control group women, over the (maximum) two year study period; and 5) to determine if successful follow-up of the study cohort was conditioned upon socio-economic status, race/ethnicity, or other factors.

In this paper, we focus on recruitment and retention of the study participants and assess whether randomization was successfully implemented. "Retention" in this paper refers to the successful completion of the formal risk assessments conducted at each visit. Subsequent reports will focus on risk factor prevalence, rate of compliance and success of each intervention as well as the overall impact of the interventions on repeat PTB.

Women who did not consent to the study were asked to a complete a brief survey. This short-form survey included questions about maternal race/ethnicity, educational attainment and income, whether or not the woman had ever been homeless, as well her insurance and marital status. The women were also asked about their height and weight, how they would rate their overall physical health, their smoking behavior during and prior to pregnancy, whether or not they had made regular dental visits prior to becoming pregnant, and if they had ever been told by a dentist or dental hygienist that they had periodontal disease. Additional information on women not consenting to the larger study but consenting to the short survey was gathered from vital statistics records including country of birth, maternal age, and gestational age of the infant. Responses to this short-form survey, in conjunction with information obtained from vital records data served as a basis for comparison of those who did as opposed to those who did not consent to the study.

To examine whether retention varied by socio-demographic and other characteristics we divided women who consented into three groups: 1) those whom we were unable to follow-up after discharge from the hospital (i.e., either because they refused or were lost to follow-up); 2) those whom we were able to locate and who completed the first one-month postpartum assessment; and 3) all other women for whom at least one additional assessment beyond the first month was completed. Comparisons among these three groups were initially completed separately for the intervention and the usual care groups. However, because the relationships were similar for both groups, we show results only for the entire study cohort.

We first conducted a series of bivariate analyses in order to document differences according to socio-demographic and other characteristics associated with recruitment (those who consented and those who did not), randomization (intervention vs. usual care group) and retention. In order to identify statistically significant relationships, p-values were generated based on appropriate statistical tests for measures of association: chi-square (nominal variables), t-values (continuous variables with only two categories), or one-way Analysis of Variance (continuous variables with more than two categories). The Bonferroni method was used to adjust p-values for multiple comparisons [27]. Characteristics which had a significantly different distribution across groups (after the Bonferroni correction) were entered into a stepwise multiple regression analysis to determine if the differences were independent of other characteristics examined. All analyses were conducted in Stata 10.1[28].

## Results

**Figure 2:**
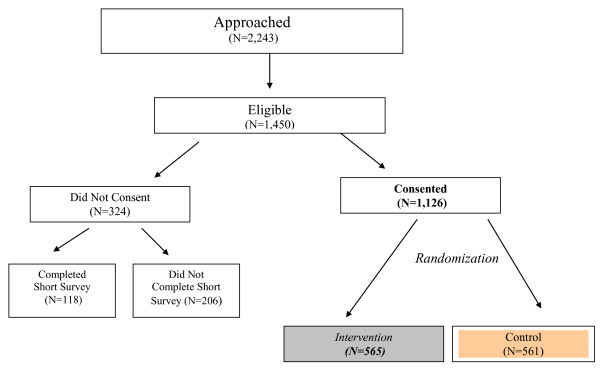
Flow Chart of Study Sample

As Figure 2 shows 2,243 women were approached and asked to participate in the study and 1,450 women (64.6%) were eligible. Of the eligible women 1,126 (77.7%) agreed to participate fully. Of the 324 who did not agree, 118 (36.4%) permitted us to administer the short-form survey described above. Of the women who agreed to participate fully in the study (n = 1,126), 561 women were randomized into the usual care and 565 women were randomized into the intervention groups.

Table 2 compares eligible women who consented and were enrolled in the study and those women who did not consent but who completed the short survey (n = 118). As we can see, those who consented differed significantly from those who did not on most characteristics. The consenting women (now deemed the study cohort), were disproportionately from minority and low SES backgrounds. Specifically, 71.5% of those who consented were African American, compared to 38.8% of those who did not. In addition, 32.1% of those who consented did not complete high school compared to only 11.9% of those who did not. Consistent with this pattern, a much higher percentage of those who consented (20.1%) compared to those who did not (6.8%) were in the lowest household income group (< $10,000 per year), and a much higher percentage (71.0 vs. 30.2%) were uninsured or on Medicaid. Consenting women were also much more likely than the non-consenting women to have reported ever having been homeless during their lifetime (14.6% vs. 0.87%) and to be unmarried at the time of delivery (81.6% vs. 47.9%). There were no significant differences between the two groups with respect to foreign-born status, Body Mass Index, overall self-reported health status, periodontal disease, smoking status prior to or during pregnancy, or presence of periodontal disease, or gestational age at birth. Finally, on average, women who consented were younger than those who did not (mean age 25.3 vs. 29.9).

**Table 2:** Socio-demographic Characteristics of Women Who Consented vs. Those Who Did Not

Characteristic	Did Not Consent Agreed to Short **Survey**^**1 **^ N = 118	Consented N = 1126	p-value	p-value*
**US born_yes**	100 (85.5)	1024 (90.9)	0.06	

				

**Race/Ethnicity**				

NH Black	46 (38.8)	805 (71.5)	0.000*	**.000**

NH-White	56 (47.4)	117 (10.4)		

Hispanic/other	16 (13.8)	204 (18.1)		

				

**Education**				

< High School	14 (11.9)	361 (32.1)	0.000*	**.000**

HS/GED	36 (30.8)	438 (38.9)		

College or more	68 (57.3)	327 (29.0)		

				

**Married at Delivery_yes**	61 (52.1)	218 (19.4)	0.000*	**.000**

				

**Household Income**			0.000*	**.000**
< =10,000	8 (6.8)	226 (20.1)		
		
10-30,000	24 (20.5)	418 (37.1)		
		
30-60,000	30 (25.6)	169 (15.0)		
		
> 60,000	38 (32.5)	93 (8.3)		
		
Don't Know/Refused	17 (14.5)	220 (19.6)		

**Insurance Status**				

Private Insurance	82 (69.8)	326 (29.0)	0.000*	**.000**

Medicaid	36 (30.2)	765 (67.9)		

Uninsured	0 (0)	35 (3.1)		

				

**Mothers Age: mean (sd)**	29.9 (5.8)	25.3 (6.4)	0.000*	**.000**

				

**Gestational Age: mean (sd)**	31.1 (3.5)	30.1 (4.0)	0.09*	

				

**Baby-alive at time of interview**				

	106 (89.7)	1004 (89.2)	0.866	

				

**Regular dental visits^2^**	90 (76.3)	659 (58.5)	0.000*	.000

				

**Periodontal disease^3^**	11 (9.6)	76 (6.8)	0.260	

				

				
		
**Physical health-fair/poor^4^**	3 (2.6)	110 (9.8)	0.02	.17

				

**Smoking before or during pregnancy**	23 (19.1)	331 (29.4)	0.02	.32

				

**Ever homeless**	1 (0.87)	164 (14.6)	0.000*	.000

				

***BMI mean (sd)***	26.3 (5.8)	26.3 (6.7)	0.971	

**^1 ^**Data only reported for those that completed the short form

**^2^**Reported making dental visits at least every 6 months

^3 ^Ever being told that they had gum disease by a dentist

**^4^**Self-Reported Physical Health fair or poor as opposed to excellent, very good or good.

*P values reflect adjustment for multiple comparisons using Bonferroni method

The results from a logistic regression analysis, with consent vs. non-consent as the dependent variable are shown in Table 3. All variables appearing in Table 2 to be significantly related to consenting to the study (after Bonferroni adjustment) were entered into the analysis. The backward elimination method was then used to reduce the number of variables to only those that met the criterion (p <.10) for remaining in the model. As we can see, race/ethnicity, household income level, marital status, and having ever been homeless, remained as significant independent predictors of participation in the study. African American women were more than five times more likely than White women to consent, and women from households with incomes less than $10,000 per year were more than 2.6 times more likely to consent than women with household incomes over $30,000. In addition, unmarried women were more than twice as likely to consent as married women, and women who reported having ever been homeless were more than 11 times more likely to consent.

**Table 3:** Results of Logistic Regression Analysis: Unadjusted and Adjusted Odds Ratios of Consenting vs. Not Consenting to Study*

	Unadjusted	Adjusted
Odds (95% C.I.)	Odds (95% C.I)	
Race/Ethnicity		
White	1.0	1.0
Hispanic/Other	5.99 (3.28, 10.93)	3.92 (2.07, 7.44)
African American	8.41 (5.42, 13.04)	5.03 (3.055, 8.39)
		
Household Income/yr^1^		
> $30,000	1.0	1.0
$10-30,000	4.51 (2.76, 7.36)	2.12 (1.20, 3.73)
< $10,000	7.33 (3.45, 15.58)	2.64 (1.17, 5.94)
		
Marital Status		
Married	1.0	1.0
Single	4.52 (3.05, 6.68)	2.10 (1.29, 3.40)
		
Ever Homeless		
No	1.0	1.0
Yes	19.75 (2.70, 140.4)	11.29 (1.54, 82.8)

^1 ^Don't Know/refused group was included in the analyses; for sake of clarity parameters are not shown.

*Selected Characteristics: variables indicating significant relationship shown in Table 2 and remaining significant after Logistic Regression using backward elimination, with p < .10 as the criterion for remaining in the final model

In Table 4 we compare characteristics of women randomized into the intervention as opposed to the control group on a similar set of characteristics as in Table 2. There were no statistically significant differences (after Bonferonni adjustment) between the intervention and control groups on any characteristic. These results are reassuring and validate the procedures used for randomization.

**Table 4:** Sociodemographic Characteristics of Women Randomized Into Control and Intervention Groups

	Intervention N = 565	Control N = 561	P-value	P-value*
**US born**	525 (92.9)	499 (88.95)	.02	**.32**
				
**Race/Ethnicity**				
NH Black	406 (71.9)	399 (71.1)	.96	
NH-White	58 (10.3)	59 (10.5)		
Hispanic/other	101 (17.9)	103 (18.4)		
				
**Education**			.62	
< HS	180 (31.9)	181 (32.3)		
HS/GED	227 (40.2)	211 (37.6)		
College or more	158 (27.96)	169 (30.1)		
				
**Married**	96 (17.0)	122 (21.9)	.04	.64
				
**Income**				
**Income **<= 10,000	113 (20)	113 (20.2)	.99	
10-30,000	212 (37.5)	205 (36.6)		
> 30,000	129 (22.8)	133 (23.7))		
DK/refused	111 (19.6)	109 (19.5)		
				
**Insurance Status**			.50	
Private Insurance	171 (30.3)	155 (27.6)		
Medicaid	374 (66.3)	390 (69.5)		
Uninsured	19 (3.4)	16 (2.9)		
				
**Age mean (sd)**	25.4 (6.5)	25.1 (6.3)	.34	
				
**GA mean (sd)**	30.2 (3.9)	30.2 (4.0)		
				
**Baby-alive**	504 (89.2)	499 (89.3)	.91	
				
**Regular dental visits**	320 (56.6)	339 (60.4)	.97	
				
**Periodontal disease**	37 (6.6)	39 (6.9)	.20	
				
**Physical health-exe/V good/good**	501 (88.7)	515 (91.8)	.79	
				
**Physical health-fair/poor**	64 (11.3)	46 (8.2)	..08	
				
**Smoking before pregnancy**	175 (30.9)	156 (27.9)	.25	
				
**Ever homeless**	88 (15.6)	76 (13.6)	.34	
				
**BMI mean (sd)**	26.4 (6.8)	26.2 (6.6)	.56	
				
**CES-D >=23**	151(26.8)	140 (25)	.50	

*P values reflect adjustment for multiple comparisons using Bonferroni method

As seen in Table 5 the first one-month postpartum assessment was completed for 83.5% (n = 472) of the intervention group (n = 565) and 76% (426) of the control group. Higher completion rates were also observed for the intervention group throughout all scheduled interviews during the follow-up period. Specifically, completion rates for the second, third, fourth and fifth postpartum assessments were 67.6% vs. 57.5%, 60.0% vs. 48.9%, 54.2% vs. 46.3% and 47.3% vs. 40.8%, for the intervention and control group women, respectively, for whom an assessment was 'due' according to the study protocols.

**Table 5:** Retention/Data Capture Rates for Study Population^1^

	*Randomization Group*		
	Intervention	Control	Total
**First Post Partum**			
**Assessment**	83.5%	76.0%	80.0%
			
**Second Post Partum**			
**Assessment**	67.6	57.5	62.6
			
**Third Post Partum**			
**Assessment**	60.0	48.9	54.4
			
**Fourth Post Partum**			
**Assessment**	54.2	46.3	50.3
			
**Fifth Post Partum**			
**Assessment**	47.3	40.8	43.6

^1 ^Denominator for calculation of rates included only those women who were expected for each assessment. Specifically, women who became pregnant again before their next assessment was due were excluded from the calculation of the above rates. In addition, as noted in the methods section, women who enrolled after 9/1/06 would not have been eligible for the full compliment of assessments; if the study period ended before a woman was a due for her next assessment, she is not included in the denominator for the calculation of the rates pertaining to that assessment.

Finally, in Table 6 we compare socio-demographic and other characteristics of the women in the study cohort for the three groups discussed above: those who consented but were lost to follow-up; those who completed only the one-month visit; and those who completed at least one additional visit beyond the first month. There are no differences among the women in the three groups. Specifically, the group of women who were lost to all follow-up (column 1) were not substantially different from women who completed only the first interview on a wide-range of socio-demographic characteristics, health behaviors, or gestational age at birth (column 2). Similarly, there were no significant differences between these women (column 2) and women for whom at least two assessments were performed (column 3) based on chi-square and T-tests including the Bonferonni correction. As noted earlier, the results shown in Table 6 are based on the sample combining the intervention and control groups as there were no differences in the results when the sample was stratified by intervention and control groups (results not shown).

**Table 6:** Socio-demographic Profile of Study Sample by Retention Status

Variable	Obtained No Information Following Randomization N = 224	Only PP1 Follow Up Survey N = 168	All else N = 734	p-value	p-value*
**US born**	200 (89.3)	152 (90.5)	672 (91.60	.57	

					

**Race/Ethnicity**					

NH Black	148 (66.1)	115 (68.5)	533 (72.6)	.34	

NH-White	30 (13.4)	18 (10.7)	74 (10.1)		

Hispanic/other	46 (20.5)	35 (20.8)	127 (17.3)		

					

**Education**					

< HS	73 (32.6)	47 (28.0)	241 (32.8)	.78	

HS/GED	84 (37.5)	69 (41.1)	285 (38.8)		

College or more	67 (29.9)	52 (30.9)	208 (28.3)		

					

**Married**	40 (17.9)	35 (20.8)	143 (19.5)		

					

**Income**					

< =10,000	32 (14.3)	36 (21.4)	158 (21.5)	.74	

10-30,000	79 (35.3)	63 (37.5)	275 (37.5)		

30-60,000	35 (15.6)	28 (16.7)	106 (14.4)		

> 60,000	19 (8.5)	10 (5.9)	64 (8.7)		

DK/refused	59 (26.3)	31(18.5)	130 (17.7)		

**Insurance Status**					

Private Insurance	66 (29.5)	38 (22.6)	222 (30.2)	.24	

Medicaid	149 (66.5)	123 (73.2)	492 (67.0)		

Uninsured	9 (4.0)	7 (4.2)	19 (2.6)		

					

**Age mean(sd)**	25.1(6.3)	25.9(6.4)	25.1(6.4)	.29	

					

**GA mean(sd)**	30.8 (3.8)	30.1(3.9)	29.9 (4.1)	02	.32

					

**Baby-alive**	203 (91.0)	152 (91.0)	648 (88.3)	.37	

					

Regular dental checkups	138 (61.1)	95(56.6)	426 (58.0)	.54	

					

Gum disease	11(4.9)	12 (7.1)	53 (7.2)	.47	

					

					
		
				.04	.66

Physical health-fair/poor	73 (32.6)	52 (30.9)	303 (41.3)		

					

Smoking before preg	70 (31.3)	48 (28.6)	213 (29.0)	.80	

					

Ever homeless	24 (10.7)	25 (14.9)	115 (15.7)	.18	

					

CES-D >=23	50(22.3)	38(22.6)	203(27.7)	.17	

					

*BMI-mean(sd)*	26.1 (7.1)	27.2 (6.5)	26.2 (6.7)	.20	


*P values reflect adjustment for multiple comparisons using Bonferroni method. Tests of significance for Age, GA, and BMI were based on one-way Analysis of Variance. All other were based on Chi-Square values

## Discussion

The recruitment rate for the PCPPP study indicated that about 78% of all women who met the study's eligibility criteria consented to participate and were successfully randomized into an intervention or usual care group. A very high percentage of minority women agreed to participate (85%) and, contrary to findings published in other reports [29,30], at considerably higher rates when compared to non-minority women (58%). In addition, independent of race/ethnicity, women residing in relatively low income households, women who reported having ever been homeless, and unmarried as opposed to married women were more likely to consent to participate.

These women fit the classic profile of those typically assumed to be less willing, or likely to volunteer for, or enroll in clinical trials. Thus, the results may be regarded as 'unexpected'. Our success is likely to be related to our recruitment protocols which were carefully designed to ameliorate and compensate for factors which might have otherwise led to disproportionate under-recruitment of minority, low SES, or other women facing especially difficult or challenging life circumstances.

Yet, it is important to note that our findings are not without precedent. For a randomized control trial designed to reduce behavioral risk factors associated with poor pregnancy outcomes conducted in Washington, D.C., El-Khorazaty et al. reported that 90% of all minority women asked to participate agreed to do so [6]. The study was limited to minority women, and so recruitment and retention rates for minority women could not be compared to those of non-minority women. However, in a systematic review of over 70 studies reporting recruitment data by race/ethnicity, Wendler et al. reported no consistent pattern of lower enrollment rates by minority status [7]. Moreover, for a subset of the 28 studies reviewed by Wendler and colleagues, involving seven surgical trials, recruitment rates were actually significantly *higher *for minority (African American and Hispanic) than for non minority (non-Hispanic White) women. Taken together with the findings presented here, this evidence suggests that minorities are at least as willing, and in some cases may even be more willing, to participate in health research.

Assumptions about the willingness of minority, as well as low income and other vulnerable populations, to participate in health studies are important to verify empirically [31-33]. Evidence of elevated risks pertaining to a wide range of chronic and acute illnesses in such populations is overwhelming and the need to reduce racial/ethnic and SES health disparities is widely recognized as a major public health priority [33-36]. Unwarranted or avoidable exclusion from randomized control trials and other legitimate health related research, of race/ethnic minorities or low income individuals, may inhibit discovery and thus the availability of effective treatments for chronic and other illnesses underpinning the persistent and pervasive SES and racial health disparities [32,33].

We found no differences in follow-up rates according to race/ethnicity, SES or other factors which might reasonably be used to define 'hard-to-reach' or 'difficult to find' populations -- again in contrast to the findings of several prior studies reporting difficulties retaining minority, low income, residentially unstable, or otherwise distressed individuals. As was the case with recruitment, every reasonable effort was made in this study to maintain the cohort, so as to overcome distrust, inconvenience, lack of resources or the presence of other factors which otherwise might have led to relatively poor follow up rates for women facing numerous barriers to participation.

It may be that, when such factors are adequately addressed, neither recruitment rates nor retention rates will necessarily be biased -- that is, compromised by under-representation of minority, low income, and other vulnerable populations. As other observers have suggested [7,8,37], it may be that the prospects for successful, reasonably proportionate recruitment and retention of 'hard to reach' or 'difficult to find' groups depends on both appropriately addressing the potential barriers and the nature of the study itself. Specifically, it may be that the perceived 'costs' in the form of inconvenience and/or reluctance in the form of suspicion of motives, are often weighed against the perceived possible 'benefits' of participation. In that light it is important to note that the PCPPP study offered a fairly broad array of what were arguably familiar and highly valued services and medical treatments, over as many as two years. The overall purpose of the intervention itself may also have been perceived to be of extremely high value by the women eligible for enrollment, especially by 'at-risk' women who may have otherwise been reluctant to agree to participate and adhere to the study protocols for ongoing assessments. At stake was the reduction of the risk of another preterm delivery, and all that implies in terms of increasing the general well being and indeed the chances of survival of a subsequent offspring.

As noted earlier, the intervention components of the study described in Table 1 were chosen based on empirical evidence of the relationship between systemic infection and the known risk factors for PTB. The implementation of the interventions were specifically designed to augment existing medical or behavioral services in the city, remove existing barriers to those services, or provide such services when generally unavailable. In all cases, all costs associated with receiving the interventions were paid for from project funds, removing any burden of cost from all study participants. Many of the services that were provided as intervention components were either not available at all in the city or not routinely available, at least at the level or depth that they were offered to the intervention group. For example, screening for BV for postpartum women is not routine practice by providers; the housing services provided - including rental assistance, and tenant/landlord mediation are well known to be difficult to find in the city; and routine screening for depression and careful case-management, counseling and referral services are also well-beyond what is available to most women, whether insured or not. It is also well known that, because the costs are so prohibitive, low income women -- whether Medicaid insured or uninsured -- typically have very limited access to dental services, especially as they pertain to the diagnosis and treatment of periodontal disease. In summary, the services associated with the interventions were clearly distinguishable from what is generally considered to be "usual care", especially where factors related to cost, inconvenience, or lack of access to care is concerned. Given that virtually all cost factors were effectively removed, that physical and other barriers pertaining the accessibility of health care services in poor neighborhoods were addressed, and that the otherwise limited scope of services provided under Medicaid were expanded, it seems plausible to suggest that low income and minority women, in particular, would indeed perceive the interventions as having considerable benefit.

The overall PCPPP follow-up rates for the first (one month) postpartum assessment of 80% are similar to those published for other studies. Retention rates for the Washington D.C. study mentioned above, for example, indicated that 79% of the enrolled women were successfully followed-up using a telephone survey at 8-10 weeks postpartum [6]. Wall et al. reported that 69% of the women randomized into a study designed to reduce postpartum smoking relapse were successfully followed-up by a telephone survey at 12 months postpartum [38]. Katz and colleagues reported a successful postpartum follow-up rate using telephone surveys at 12 months for their study, involving a multi-site parenting intervention, of 59% [5]. Postpartum follow up rates for similar studies conducted in other countries with a universal system of care, however, appear to be somewhat better. In postpartum smoking relapse prevention RCT in Canada, for example, follow-up rates were reported to be 95% at 12 months [39].

In the ongoing, large scale Fragile Families observational study, which recruited subjects from 20 major U.S. cities, and involved a national sample of more than 4,700 index births, 89% and 86% of families were successfully followed-up by telephone survey at one and three year time points, respectfully [40]. However, follow-up rates for the in-home assessment portion of the study were considerably lower. According to the published data, only 54% of the intended in-home assessments at three years (2,581 of the 4,789 baseline sample) were successfully completed. This would appear to be a somewhat higher rate of successful follow-up than reported here (47.3% of the expected final postpartum PCPPP assessments were completed). The lower rate for the PCPPP assessments presented here may be attributable to the assessments for the intervention and usual care group requiring on-site clinical visits, rather than simple follow-up via a telephone survey or home visit.

Although the financial incentives for ongoing participation for both groups was equal, and both groups were afforded the same accommodations and reimbursements associated with all on-site clinic assessments, assessments pertaining to all time periods were completed at higher rates by the intervention compared to the usual care group. Because, by definition, the groups were randomized, these differences are unlikely to be caused by factors other than the nature of the intervention itself. Consequently, the results are consistent with the notion that the extent and nature of the medical and behavior services constituting the PCPPP intervention arm were highly valued and, in and of themselves, served as an incentive for the intervention group to remain in the study.

With regard to the comparison of women who consented vs. those who did not, it is important to note that, of all those who did not consent, only 36% (n = 118) agreed to complete the short form survey. Hence, the 'true' characteristics of all the women who did not consent were estimated based on this subsample. As with any subsample which is not randomly selected it was subject to selection bias. In theory, the bias could have resulted in a disproportionately greater number of higher income, more educated, non-minority women having completed the short-form survey. In that case what appeared to be evidence of greater willingness on the part of minority and low SES women to participate in the larger study could instead be a reflection of selection bias with respect to the characteristics of the subsample of women who agreed to complete the short-form survey. To address this concern we conducted a supplementary analysis of birth certificate data for all women who delivered preterm infants at the hospitals where women were recruited, during the same time period active recruitment occurred. The distributions for age, marital status, education, and race/ethnicity calculated using these data were virtually identical to those we calculated for the combined data gathered from all women approached at delivery - that is, for both those who completed the short-form survey and those who consented to the larger study. If there were any substantial selection bias related to sociodemographic factors we would expect these distributions to be discernibly different, with the data from birth records reflecting the 'unusual' group who declined the short-form survey. Since the distributions were not different, we concluded that the data from the short-form surveys were representative of all non-consenting women, and thus that differences observed between those who consented and those who did not were not the result of any selection bias.

## Conclusions

In summary the findings from the analysis of recruitment, randomization and retention data presented here adds to a small but growing body of literature which increasingly challenges a widely held belief that low income and minority women are necessarily averse to enrolling in clinical trials or community studies. This study demonstrates that if the barriers to participation of disadvantaged populations are appropriately addressed, high participation rates can be achieved. Much of the concern about the under-participation in randomized control trials and community studies by racial/ethnic minorities, in particular, has focused on the past abuses or ethical lapses pertaining to inadequately informing (or in some cases deliberately misleading) potential subjects about potential risks [8,37,41]. In no way would we wish to suggest that such concerns are unwarranted, or that they need not be carefully and fully addressed. Rather, the important point is that these concerns should be weighed in the context of the real and perceived benefits of the study interventions and findings to both the individuals involved and to the communities where they live.

## List of Abbreviations

BMI: (body mass index); RCT: (Randomized clinical trial); PCPPP: (Philadelphia Collaborative Preterm Prevention Project); PTB: (preterm birth);, SI: (Systemic Inflammation);

## Competing interests

The authors declare that they have no competing interests.

## Authors' contributions

**DAW **directed the analyses for this study and was primarily responsible for manuscript preparation. **JFC **was the principal investigator of the Project, conceived the intervention trial, contributed to the design and methodology of this study , supervised the overall study conduct, and assisted in manuscript preparation. **JCC, RLG and VKH and ITE **contributed to the design and methodology of the study and assisted in manuscript preparation. **JRB, IMB, and EFD **conducted literature reviews and contributed to preparation of the tables and assisted in manuscript preparation and editing. **LM **assumed primary responsibility for all data analyses and assisted in the preparation of the manuscript. All authors read and approved the final manuscript.

## Pre-publication history

The pre-publication history for this paper can be accessed here:

http://www.biomedcentral.com/1471-2288/10/88/prepub
